# Prognostic Value of the AST/ALT Ratio in Patients with Septic Shock: A Prospective, Multicenter, Registry-Based Observational Study

**DOI:** 10.3390/diagnostics15141773

**Published:** 2025-07-14

**Authors:** Sungwoo Choi, Sangun Nah, Gil Joon Suh, Sung-Hyuk Choi, Sung Phil Chung, Won Young Kim, Tae Ho Lim, Sangchun Choi, Tae Gun Shin, Sangsoo Han

**Affiliations:** 1 Department of Emergency Medicine, Soonchunhyang University Bucheon Hospital, Bucheon 14584, Republic of Korea; csw3613@naver.com (S.C.); potter325@naver.com (S.N.); avenue5933@gmail.com (S.C.); 2 Department of Emergency Medicine, Seoul National University Hospital, Seoul 03080, Republic of Korea; suhgil@snu.ac.kr; 3 Department of Emergency Medicine, Korea University Guro Hospital, Seoul 08308, Republic of Korea; kuedchoi@korea.ac.kr; 4 Department of Emergency Medicine, Gangnam Severance Hospital, Yonsei University College of Medicine, Seoul 06273, Republic of Korea; emstar@naver.com; 5 Department of Emergency Medicine, University of Ulsan College of Medicine, Asan Medical Center, Seoul 05505, Republic of Korea; wonpia73@naver.com; 6 Department of Emergency Medicine, College of Medicine, Hanyang University, Seoul 15495, Republic of Korea; erthim@gmail.com; 7 Department of Emergency Medicine, Samsung Medical Center, Sungkyunkwan University School of Medicine, Seoul 06351, Republic of Korea

**Keywords:** sepsis, aspartate aminotransferase, alanine aminotransferase, Sequential Organ Failure Assessment score

## Abstract

**Background/Objectives**: Sepsis is a leading cause of mortality. The AST/ALT ratio may serve as a valuable marker for prediction in patients with various diseases. This study analyzed the prognostic value of this ratio in patients with sepsis. **Methods**: A retrospective analysis was performed on data from a prospective registry of septic shock patients, collected across multiple centers from October 2015 to December 2022. The main outcome of interest was mortality within 28 days. We evaluated the predictive accuracy of 28-day mortality for variables with the Sequential Organ Failure Assessment (SOFA) score, aspartate transaminase (AST) levels, alanine transaminase (ALT) levels, the AST/ALT ratio, and the combination of the SOFA + AST/ALT ratio using the area under the receiver operating characteristics curve (AUROC). A Kaplan–Meier curve was used to compare the 28-day mortality between the AST/ALT subgroups (≥1.84 and <1.84). Stepwise multivariable Cox proportional hazards analyses were performed to determine the association between 28-day mortality and an AST/ALT ratio ≥ 1.84. **Results**: The AST/ALT ratio had a significantly higher discriminatory ability for predicting 28-day mortality compared to either AST or ALT. In addition, combining the AST/ALT ratio with the SOFA score improved the predictive accuracy compared to the SOFA alone. A multivariable Cox regression analysis demonstrated that an AST/ALT ratio ≥ 1.84 was associated with a higher risk of death within 28 days. **Conclusions**: The AST/ALT ratio at emergency department admission in sepsis patients is associated with 28-day mortality and, when combined with the SOFA score, provides additional prognostic information with moderate accuracy.

## 1. Introduction

Sepsis is a life-threatening state caused by a dysregulated response of the host to infection. When it is accompanied by circulatory and cellular/metabolic abnormalities, it is classified as septic shock, which often results in increased mortality [1]. Sepsis remains one of the significant contributors to mortality in emergency departments (EDs) and intensive care units (ICUs) [2,3]. In 2017, there were an estimated 48.9 million new cases of sepsis globally, resulting in approximately 11 million deaths. This represented 19.7% of all deaths worldwide [4].

Sepsis-associated organ dysfunction is commonly assessed using the Sequential Organ Failure Assessment (SOFA) score. This scoring system evaluates clinical indicators such as the mean arterial pressure (MAP), urine output, Glasgow Coma Scale (GCS), and laboratory test results, including the PaO2, platelet count, and levels of creatinine and bilirubin [1,5]. A SOFA score of 2 or more points is linked to a hospital mortality rate greater than 10% [1]. Among the organs assessed by this scoring system, the liver (the largest gland in the body) plays a critical role in maintaining metabolic and immune system homeostasis [6]. In particular, when complications such as liver dysfunction or failure occur as a result of sepsis, they can significantly influence the prognosis and mortality of the disease [6,7].

One factor linked to liver damage is the aspartate aminotransferase (AST)/alanine aminotransferase (ALT) ratio, first introduced by Fernando De Ritis in 1957. The ratio is correlated with various liver diseases [8,9] and is significantly associated with several conditions, including malignant tumors, stroke, myocardial infarction, limb ischemia, renal failure, and respiratory failure, serving as both a prognostic and predictive marker of poor outcomes [10,11,12,13,14]. Furthermore, it may be a valuable prognostic factor in patients with sepsis [15,16]. However, most studies have been single-center investigations with relatively small sample sizes [15,16].

Therefore, in this study, we included a large cohort of patients from multiple centers to analyze whether the AST/ALT ratio offers meaningful prognostic value in sepsis patients and whether these findings can be generalized.

## 2. Materials and Methods

### 2.1. Study Design

Using a dataset derived from the multicenter Korean Shock Society (KoSS) registry, which prospectively collected data between October 2015 and December 2022, we conducted a retrospective observational study. The KoSS operates as a nationwide collaborative network aimed at enhancing sepsis diagnosis and treatment strategies. A prospective registry was initiated in October 2015 to collect standardized data on the septic shock patients seen at the EDs of 12 university-affiliated hospitals in South Korea [17,18,19]. The eligible participants were adults aged over 18 years who had either suspected or confirmed infection, along with signs of hypoperfusion or refractory hypotension [20]. As the implementation of the KoSS registry began before the publication of the Sepsis-3 criteria, this inclusion approach was based on the refractory sepsis-induced hypotension or tissue hypoperfusion definition in the 2012 Surviving Sepsis Campaign (SSC) guidelines and was consistently maintained throughout the study period to ensure uniform enrollment [17,21,22]. Hypoperfusion was determined by a lactate level of 4 mmol/L or higher, while refractory hypotension referred to ongoing low blood pressure (MAP < 70 mmHg, SBP < 90 mmHg, or a reduction in SBP > 40 mmHg) following intravenous (IV) fluid replacement (20–30 mL/kg or ≥1 L crystalloid solution) or the need for vasopressor support despite fluid administration [22]. In addition, we separately identified patients who fulfilled the Sepsis-3 criteria of septic shock for the analysis. Septic shock based on the Sepsis-3 criteria was defined as sepsis with sustained hypotension requiring vasopressors to maintain an MAP ≥ 65 mmHg and a serum level of lactate > 2 mmol/L [1]. In addition, all patients were enrolled at the ED upon their initial presentation, prior to any ICU admission.

The exclusion criteria included patients with missing data, those transferred to another hospital, those with do-not-resuscitate orders, those lost to follow-up, and those with liver disease. The ethical approval for this study was granted by the institutional review boards (IRBs) of each participating institution, and informed consent was waived by the IRBs of each participating institution in accordance with ethical regulations. Although the KoSS registry is an ongoing prospective database, this study included only patients enrolled up to December 2022, in accordance with the IRB-approved protocol.

### 2.2. Data Collection

The KoSS registry includes standardized definitions for 200 variables [23]. The coordinator of each hospital anonymized and gathered data employing standardized web-based reporting forms. The patient enrollment and data collection were carried out jointly by the attending emergency physicians and trained research coordinators at each site. To maintain data integrity, the system automatically screened for extreme or erroneous values. A quality control committee, consisting of emergency medicine specialists, investigators, and regional research coordinators from each participating hospital, was established to consistently monitor and assess the quality of the data.

The following data were extracted from the registry: baseline clinical characteristics, comorbidities, vital signs, severity scores (SOFA score and Acute Physiology and Chronic Health Evaluation [APACHE] II upon enrollment), suspected infection, laboratory findings (white blood cells, hemoglobin, platelet count, sodium, chloride, potassium, creatinine, C-reactive protein [CRP], blood urea nitrogen, procalcitonin, lactate, international normalized ratio [INR], pH, albumin, alanine transaminase [ALT], aspartate transaminase [AST], and AST/ALT ratio), interventions (including renal replacement therapy [RRT], steroid use, and mechanical ventilation [MV]), and outcomes. The laboratory results were based on the initial findings upon the patients’ ED arrival.

The APACHE II score used in this study was the lowest score within 24 h of the ED arrival, and the SOFA score was recorded upon the registry enrollment. Mortality within 28 days served as the primary outcome, whereas secondary outcomes included mortality within 90 days, ICU admission, MV, and RRT within 24 h.

### 2.3. Statistical Analysis

The continuous variables were summarized as the median and interquartile range (IQR) or the mean and standard deviation, based on the Shapiro–Wilk test results. The categorical variables were presented as counts (percentages) and analyzed using the Fisher’s exact test or the χ^2^ test. The continuous variables were compared using either the Mann–Whitney U test or the *t*-test.

We evaluated the AST/ALT ratio, which has been suggested to be a predictor of mortality in sepsis in recent studies, by comparing it with the SOFA score [15,16]. The discriminatory capacity for 28- and 90-day mortality was assessed by comparing the area under the receiver operating characteristic curve (AUROC) for each variable (SOFA, AST, ALT, and AST/ALT ratio) and in combination with the SOFA and the AST/ALT ratio. Specific AUROC values with 95% confidence intervals (CIs) were determined. By using Youden’s index, we identified the optimal cut-off value for the AST/ALT ratio. Based on this cut-off (≥1.84 and <1.84), the patients were grouped into two subgroups.

Kaplan–Meier curves were generated to compare the 28- and 90-day mortality between the AST/ALT subgroups. A stepwise multivariable Cox proportional hazards regression was conducted to assess the association between the AST/ALT ratio (≥1.84) and the primary and secondary outcomes. The proportional hazards assumption for the Cox analysis was evaluated through Schoenfeld residuals. These analyses were also performed in the subgroup of patients with septic shock, as defined by the Sepsis-3 criteria. A two-tailed *p*-value of less than 0.05 was interpreted as indicating statistical significance.

We used R software (version 4.2.2, The R Foundation, Vienna, Austria) and Python (version 3.12), with libraries such as NumPy (version 2.0.2), Pandas (version 2.2.2), Matplotlib (version 3.10.0), and Seaborn (version 0.13.2) for the data visualization, manipulation, and statistical testing.

## 3. Results

**Figure 1:**
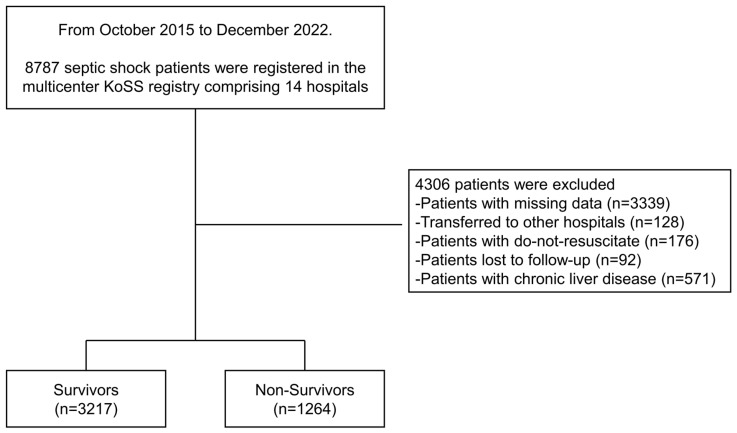
Study flow of patient inclusion and exclusion criteria.

In total, 8787 subjects were enrolled in the KoSS registry between October 2015 and December 2022. Of these, 4306 patients were excluded due to missing data, 128 were transferred to other hospitals, 176 had do-not-resuscitate orders, 92 were lost to follow-up, and 571 had chronic liver disease. Finally, 4481 patients were included in this study (Figure 1).

The baseline characteristics of the 28-day survivor and non-survivor groups are summarized in Table 1. The 28-day mortality rate was 28.2% (*n* = 1264). The non-survivor group was older than the survivor group (72 years vs. 69 years, *p* < 0.01), and the proportion of females was lower in the non-survivor group compared to the survivor group (40.7% vs. 44.2%, *p* = 0.01). Across all patients, hypertension was the most frequent comorbidity.

As shown in Table 2, the proportion of patients with septic shock based on the Sepsis-3 criteria was more frequent in the 28-day non-survivor group (79.1% vs. 59.5%, *p* < 0.01). Regarding the sepsis-related scores, the SOFA score (median 8 vs. 5, *p* < 0.01) and the APACHE II score (median 26 vs. 19, *p* < 0.01) were significantly higher in the non-survivor group. The most prevalent infection source in that group was respiratory infection (36.1%), while intra-abdominal infection was most frequent in the survivor group (33.9%). In the non-survivor group, increased serum levels of lactate (5.2 vs. 2.9 mmol/L, *p* < 0.01), CRP (17.3 vs. 13.8 mg/dL, *p* < 0.01), and the AST/ALT ratio (1.91 vs. 1.47, *p* < 0.01) were observed. In addition, the use of MV and RRT within 24 h was significantly higher in the non-survivor group vs. the survivor group (53.6% vs. 15% and 24.9% vs. 5.8%, respectively; *p* < 0.01).

**Figure 2:**
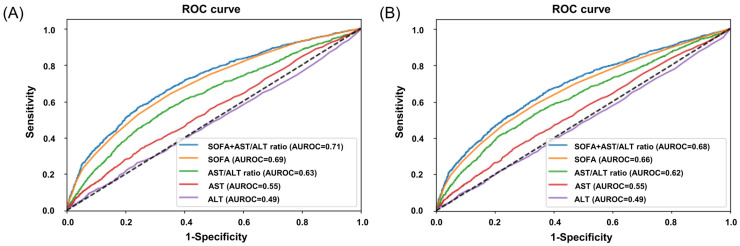
The ROC curves for 28- and 90-day mortality. (**A**) The ROC curves for 28-day mortality. The AUROC for the SOFA + AST/ALT ratio is 0.71 (95% CI, 0.69–0.73, *p* < 0.01), SOFA is 0.69 (95% CI, 0.68–0.71, *p* < 0.01), AST/ALT ratio is 0.63 (95% CI, 0.61–0.65, *p* < 0.01), AST is 0.55 (95% CI, 0.53–0.57, *p* < 0.01), and ALT is 0.49 (95% CI, 0.47–0.51, *p* = 0.86). (**B**) The ROC curves for 90-day mortality. The AUROC for the SOFA + AST/ALT ratio is 0.68 (95% CI, 0.67–0.70, *p* < 0.01), SOFA is 0.66 (95% CI, 0.65–0.68, *p* < 0.01), AST/ALT ratio is 0.62 (95% CI, 0.60–0.64, *p* < 0.01), AST is 0.55 (95% CI, 0.53–0.56, *p* < 0.01), and ALT is 0.49 (95% CI, 0.47–0.50, *p* = 0.63).

The discrimination of 28-day mortality was significantly higher using the AST/ALT ratio (AUROC, 0.62; 95% CI, 0.61–0.64) compared to either AST (AUROC, 0.56; 95% CI, 0.54–0.58) or ALT (AUROC, 0.50; 95% CI, 0.48–0.52) (Figure 2A). Furthermore, when combining the AST/ALT ratio with the SOFA score, the AUROC for the SOFA + AST/ALT ratio (AUROC, 0.71; 95% CI, 0.70–0.73) was greater than for the SOFA score alone (AUROC, 0.69; 95% CI, 0.68–0.71). A similar trend was observed for 90-day mortality (Figure 2B).

Table 3 presents the hazard ratios for both the primary and secondary outcomes, derived from univariable and multivariable Cox proportional hazards models. In the multivariable analysis, the AST/ALT ratio ≥ 1.84 was significantly associated with 28-day mortality (adjusted HR, 1.46; 95% CI, 1.30–1.64; *p* < 0.01), 90-day mortality (adjusted HR, 1.33; 95% CI, 1.20–1.46; *p* < 0.01), MV within 24 h (adjusted HR, 1.28; 95% CI, 1.13–1.43; *p* < 0.01), and RRT within 24 h (adjusted HR, 1.50; 95% CI, 1.25–1.80; *p* < 0.01). However, no significant association was found with ICU admission (adjusted HR, 1.01; 95% CI, 0.91–1.11; *p* = 0.85).

**Figure 3:**
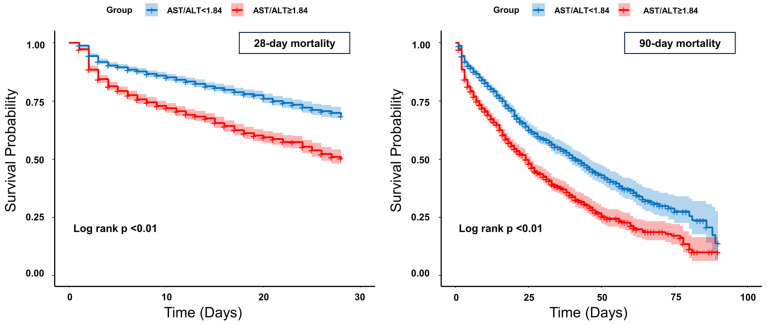
Kaplan–Meier curves for 28-day and 90-day mortality.

Figure 3 shows the survival probability for 28-day and 90-day mortality below and above the cut-off point of the AST/ALT ratio. An AST/ALT ratio ≥ 1.84 was significantly associated with increased 28-day and 90-day mortality (log-rank test, *p* < 0.01). In the subgroup of patients with septic shock (Sepsis-3 definition), the results of the Cox regression (Table S1), ROC curves (Figure S1), and Kaplan–Meier curves (Figure S2) for 28- and 90-day mortality were comparable to those of the overall cohort (refer to the Supplementary Materials).

## 4. Discussion

We analyzed the AST/ALT ratio in sepsis patients and found that an AST/ALT ratio ≥ 1.84 is associated with both 28- and 90-day mortality. Furthermore, combining the AST/ALT ratio with the SOFA score improved the prognostic value, although the predictive accuracy of both the AST/ALT ratio alone and in combination with the SOFA score remained moderate. Previous studies have evaluated various tools for predicting mortality, including the Modified Early Warning Score, quick Sequential Organ Failure Assessment, National Early Warning Score, and Universal Vital Assessment. Depending on the study cohort, these tools have demonstrated poor to moderate predictive performance for mortality [24,25,26]. In this context, the moderate performance of the combined AST/ALT ratio and SOFA score could still be valuable for diagnosis and prognosis prediction in sepsis patients. Our findings align with earlier single-center studies that proposed AST/ALT ratio cut-off values of 1.84 and 1.22 for predicting sepsis outcomes [15,16]. To the best of our knowledge, this is the first multi-center study with a large patient cohort to analyze this association.

The liver is essential for a wide range of physiological functions, including detoxification, storage, energy and nutrient regulation, and coagulation, which makes it a key organ in metabolic and immune processes. In sepsis patients, the liver may be damaged by pathogens, toxins, and inflammatory substances, leading to hepatocellular dysfunction, characterized by impaired synthetic and clearance functions. Sepsis-induced liver injury is driven by multiple interconnected mechanisms, including systemic inflammation, oxidative stress, mitochondrial dysfunction, and microvascular hypoperfusion [27]. The activation of Toll-like receptors and NOD-like receptors on Kupffer cells by pathogen-associated molecular patterns leads to the massive production of pro-inflammatory cytokines, resulting in hepatocyte apoptosis and necrosis [27,28]. In addition, oxidative stress, mitochondrial damage, hypoxic hepatitis due to circulatory failure, and bacterial translocation via the gut–liver axis also play critical roles in hepatic injury during sepsis [27,29]. These intertwined processes not only contribute to liver dysfunction but also correlate with increased disease severity and mortality. These damages can result in irreversible hepatocyte injury, contributing to overall liver dysfunction [6]. Such hepatocellular damage leads to the release of AST and ALT into the bloodstream, causing elevated levels of these enzymes. In septic patients, the high metabolic demands for nutrients and oxygen can induce tissue and cellular hypoxia, potentially leading to microcirculatory collapse and tissue ischemia in the liver [16,30].

In a healthy state, the liver cell turnover and enzyme removal from the plasma are balanced. The hepatic AST to ALT ratio is typically approximately 2:1. Given that the half-lives of AST and ALT are 18 and 36 h, respectively, this ratio remains relatively stable. AST is more involved in aerobic glycolysis, while ALT participates in the glucose–alanine cycle and anaerobic metabolism. However, this balance can be disrupted by various factors, including viral hepatitis, alcoholic liver disease, and muscle disorders [8]. Recent studies have suggested that an increased AST/ALT ratio can help predict risk and mortality in cancer patients [31,32,33,34,35]. This increase in cancer is reportedly linked to the Warburg effect, where cancer cells preferentially undergo aerobic glycolysis, increasing the glucose uptake and lactate production even in the presence of oxygen [36]. Similarly, in patients with sepsis, immune system activation causes immune cells like macrophages, dendritic cells, and T cells to shift from oxidative phosphorylation to aerobic glycolysis [37]. Previous studies have also indicated that an elevated AST/ALT ratio is helpful in predicting the prognosis of sepsis patients [15,16]. Our findings suggest that using the AST/ALT ratio offers better discrimination of mortality risk in sepsis patients.

In addition, while ALT is relatively specific to the liver, AST is also found in other tissues, including the heart, skeletal muscle, brain, and kidneys [10,38]. Therefore, an elevated AST/ALT ratio may reflect not only hepatic oxidative stress and mitochondrial injury but also multi-organ damage [39,40,41]. Previous studies have reported that a higher AST/ALT ratio correlates with increased levels of inflammatory markers [42,43]. Systemic inflammatory responses, oxidative stress, mitochondrial dysfunction, and multiple organ failure are all common features observed in patients with severe sepsis [44]. Thus, an elevated AST/ALT ratio may serve as a potential predictor of poor prognosis in sepsis patients.

In this study, it was confirmed that using both the SOFA score and the AST/ALT ratio together provides better discrimination of mortality than using the SOFA score alone. The SOFA score incorporates various clinical indicators, such as the MAP, GCS, and urine output, along with laboratory test results such as the PaO2, platelet count, creatinine, and bilirubin, to provide a comprehensive assessment of organ function [1]. Among these, bilirubin serves as an indicator of liver damage [1]. Liver damage due to hypoxemia or hypoperfusion caused by sepsis affects bile synthesis, leading to elevated total bilirubin levels [45]. However, liver failure resulting from sepsis does not always lead to simultaneous increases in the serum levels of bilirubin and the AST/ALT ratio [6,46,47]. Therefore, although the SOFA score does not account for the AST/ALT ratio, incorporating this ratio, which can reflect liver dysfunction, may improve the ability to discriminate mortality in sepsis patients, as suggested by our results.

In this study, a cut-off value of 1.84 for the AST/ALT ratio was associated with an increased risk of mortality. Similar findings have been reported in previous studies. For example, one study that analyzed 30-day mortality in sepsis patients suggested a cut-off value of 1.8 [15], while another study, which analyzed 180-day mortality in sepsis patients, proposed a cut-off of 1.22 [16]. This variation in cut-off values is likely due to differences in the time frame of mortality and the study populations, with one study focusing on internal ICU patients and the other on surgical ICU patients. However, the cut-off value of 1.84 identified in this study, which analyzed all sepsis patients admitted to the ED and considered both 28-day and 90-day mortality, appears to be meaningful. In addition, the AST/ALT ratio can increase to ≥2.0 in liver-related conditions such as acute viral hepatitis, alcoholic hepatitis, or fulminant hepatitis [8]. Considering the cut-off values reported in previous sepsis-related studies and the extent of the ratio increases seen in other liver diseases, the 1.84 cut-off value suggested by this study is likely to be clinically significant for the management of sepsis.

This work is significant for the analysis of sepsis and the AST/ALT ratio, enrolling a large cohort of patients across multiple centers. However, several limitations should be considered. First, we did not assess dynamic changes in the AST/ALT ratio during the hospital stay. Nevertheless, the analysis of initial test results at the time of the ED visit provided meaningful insights, suggesting that early ED values can be helpful for prognosis prediction. Future research is needed to examine the dynamic changes in the ratio over time. Second, the exclusion of 3735 patients (42.5%) due to missing data raises the possibility of selection bias. These excluded patients may have differed in their clinical severity or treatment patterns, potentially limiting the generalizability of our findings. Future studies should consider minimizing missing data and evaluating the impact of such exclusions. Third, while our study included data from multiple centers and a large cohort, it was conducted in a single country, which limits the applicability of our findings to populations in other countries or with different ethnic groups. As a result, the generalizability of the findings to other populations may be restricted. To address this, additional large-scale studies involving diverse ethnicities and countries are required, taking these factors into account.

## 5. Conclusions

A higher AST/ALT ratio at the time of the ED visit in sepsis patients is associated with 28-day and 90-day mortality. Combining the AST/ALT ratio with the SOFA score improves the prognostic value, although the predictive accuracy of both the AST/ALT ratio alone and in combination with the SOFA score remains moderate. Given the multicenter design and large sample size, our findings may have potential generalizability to broader emergency department populations; however, this should be interpreted with caution due to the high rate of patient exclusions and the study’s limitation to a single country and ethnic group.

## Figures and Tables

**Table 1:** Comparison of clinical characteristics based on 28-day mortality outcomes.

	Survival (*n* = 3217)	Non-Survival (*n* = 1264)	*p*-Value
Age, years	69 [60–77]	72 [63–80.25]	<0.01
Female, *n* (%)	1423 (44.2)	514 (40.7)	0.03
Initial vital signs at ED arrival			
Systolic BP, mmHg	92 [78–113]	91 [76–115]	0.29
Diastolic BP, mmHg	56 [48–67]	56 [46–69]	0.38
Body temperature, °C	37.9 [36.9–38.8]	37.1 [36.3–38.1]	<0.01
Respiratory rate, /min	20 [18–23]	22 [20–28]	<0.01
Heart rate, beats/min	110 [93–128]	113 [96–130]	<0.01
Comorbidities, *n* (%)			
Diabetes mellitus	1053 (32.7)	424 (33.5)	0.63
Hypertension	1377 (42.8)	594 (47.0)	0.01
Malignancy	966 (30)	460 (36.4)	<0.01
Cardiovascular disease	441 (13.7)	211 (16.7)	0.01
Chronic lung disease	217 (6.8)	132 (10.4)	<0.01
Chronic kidney disease	272 (8.5)	132 (10.4)	0.04

Note: Values are expressed as numbers (proportions) or medians [interquartile ranges]. Abbreviations: BP, blood pressure; ED, emergency department.

**Table 2:** Septic shock-related data and laboratory findings according to 28-day mortality.

	Survival (*n* = 3217)	Non-Survival (*n* = 1264)	*p*-Value
Septic shock (Sepsis-3 definition), *n* (%)	1914 (59.5)	1000 (79.1)	<0.01
SOFA score	5 [4–7]	8 [5–10]	<0.01
APACHE II score	19 [14–25]	26 [20–34]	<0.01
Infection source, *n* (%)			<0.01
Intra-abdominal infection	1090 (33.9)	283 (22.4)	
Urinary tract infection	680 (21.1)	129 (10.2)	
Respiratory tract infection	611 (19)	456 (36.1)	
Other	836 (26)	396 (31.3)	
Laboratory findings			
White blood cells, ×10^3^/mm^3^	10,000 [4200–16,600]	8800 [2300–17,112.5]	<0.01
Platelet, ×10^3^/mm^3^	141 [83–218]	120 [48–223]	<0.01
Hemoglobin, g/dL	10.8 [9.1–12.5]	10.1 [8.6–12]	<0.01
Sodium, mmol/L	135 [131–138]	135 [131–139]	<0.01
Potassium, mmol/L	4 [3.6–4.6]	4.3 [3.7–5]	<0.01
Chloride, mmol/L	100 [96–104]	100 [95–105]	0.12
Creatinine, mg/dL	1.36 [0.94–2.12]	1.63 [1.06–2.62]	<0.01
Blood urea nitrogen, mg/dL	26.8 [18.4–39.4]	36.5 [24.1–54]	<0.01
CRP, mg/dL	13.8 [5.8–23.5]	17.3 [8.5–27.9]	<0.01
Procalcitonin, ng/mL	8.92 [1.43–36.24]	10.41 [1.65–41.48]	0.15
Lactate, mmol/L	2.9 [1.7–4.9]	5.2 [3–8.2]	<0.01
INR	1.22 [1.11–1.38]	1.36 [1.2–1.62]	<0.01
pH	7.44 [7.39–7.48]	7.39 [7.28–7.46]	<0.01
Albumin, g/dL	3.1 [2.7–3.5]	2.7 [2.2–3.12]	<0.01
AST/ALT ratio	1.47 [1.07–2.07]	1.91 [1.3–2.78]	<0.01
AST, U/L	39 [24–78]	45 [26–114]	<0.01
ALT, U/L	26 [15–55]	26 [14–59]	0.85
Duration of hospitalization, d	15 [9–25]	5 [2–12]	<0.01
Steroid use, *n* (%)	825 (25.7)	537 (42.5)	<0.01
Admission to the ICU, *n* (%)	1736 (54)	876 (69.3)	<0.01
MV within 24 h, *n* (%)	482 (15)	678 (53.6)	<0.01
RRT within 24 h, *n* (%)	287 (5.8)	302 (24.9)	<0.01

Note: Values are presented as medians [interquartile ranges] or numbers (proportions). Abbreviations: qSOFA, quick sepsis-related organ failure assessment; SOFA, sepsis-related organ failure assessment; APACHE, acute physiology and chronic health evaluation; AST, aspartate transaminase; ALT, alanine transaminase; CRP, C-reactive protein; ICU, intensive care unit; MV, mechanical ventilation; RRT, renal replacement therapy.

**Table 3:** The univariable and multivariable Cox regression analyses of the AST/ALT ratio for predicting primary and secondary outcomes.

	Univariable		Multivariable	
	Unadjusted HR (95% CI)	*p*-Value	Adjusted HR (95% CI)	*p*-Value
Primary outcome				
28-day mortality	1.95 (1.75–2.18)	<0.01	1.46 (1.30–1.64)	<0.01
Secondary outcome				
90-day mortality	1.68 (1.53–1.85)	<0.01	1.33 (1.20–1.46)	<0.01
Admission to the ICU	0.89 (0.81–0.96)	0.01	1.01 (0.91–1.11)	0.85
MV within 24hrs	1.59 (1.42–1.79)	<0.01	1.28 (1.13–1.43)	<0.01
RRT within 24hrs	1.5 (1.26–1.81)	<0.01	1.50 (1.25–1.80)	<0.01

Abbreviations: HR, hazard ratio; MV, mechanical ventilation; ICU, intensive care unit; RRT, renal replacement therapy.

## Data Availability

The dataset supporting the findings of this study is available upon reasonable request from the corresponding authors (Tae Gun Shin, drshin88@gmail.com; Tel.: +82-2-3410-2053 and Sangsoo Han, brayden0819@daum.net; Tel.: +82-32-621-5116) or the Korean Shock Society (Koss) (59club@daum.net) upon reasonable request.

## Supplementary Materials

The following supporting information can be downloaded at: https://www.mdpi.com/article/10.3390/diagnostics15141773/s1, Table S1: Univariable and multivariable Cox regression analyses of the AST/ALT ratio for predicting primary and secondary outcomes in Sepsis-3-defined septic shock; Figure S1: ROC curves for 28- and 90-day mortality in septic shock based on the Sepsis-3 criteria; Figure S2: Kaplan-Meier curves for 28-day and 90-day mortality in septic shock based on the Sepsis-3 criteria.

## Abbreviations

The abbreviations listed below are used in this paper:

AST	Aspartate Transaminase
ALT	Alanine Transaminase
SOFA	Sequential Organ Failure Assessment
APACHE II	Acute Physiology and Chronic Health Evaluation II
CRP	C-reactive Protein
INR	International Normalized Ratio
MV	Mechanical Ventilation
RRT	Renal Replacement Therapy
ED	Emergency Department
ICU	Intensive Care Unit
KoSS	Korean Shock Society
MAP	Mean Arterial Pressure
GCS	Glasgow Coma Scale
IV	Intravenous
HR	Hazard Ratio
AUROC	Area Under the Receiver Operating Characteristics Curve
IQR	Interquartile Range
IRB	Institutional Review Board
SBP	Systolic Blood Pressure
CI	Confidence Interval
