# Supplementary material for: Prognostic Value of the AST/ALT Ratio in Patients with Septic Shock: A Prospective, Multicenter, Registry-Based Observational Study
Source: Diagnostics (Basel). 2025 Jul 14;15(14):1773. doi: 10.3390/diagnostics15141773 (PMC12293526; doi:10.3390/diagnostics15141773)

Table S1. Univariable and multivariable Cox regression analyses of the AST/ALT ratio for predicting primary and secondary outcomes in Sepsis-3-defined septic shock.

	**Univariable**		**Multivariable**	
	**Unadjusted HR (95% CI)**	***p*-value**	**Adjusted HR (95% CI)**	***p*-value**
Primary outcome				
28-day mortality	1.82 (1.61-2.06)	<0.01	1.48 (1.31-1.69)	<0.01
Secondary outcome				
90-day mortality	1.65 (1.48-1.84)	<0.01	1.39 (1.24-1.56)	<0.01
Admission to the ICU	0.99 (0.87-1.13)	0.90	1.06 (0.93-1.22)	0.38
MV within 24hrs	1.55 (1.37-1.76)	<0.01	1.34 (1.18-1.52)	<0.01
RRT within 24hrs	1.85 (1.53-2.23)	<0.01	1.57 (1.29-1.90)	<0.01

Abbreviations: HR, hazard ratio; MV, mechanical ventilation; ICU, intensive care unit; RRT, renal replacement therapy.

Figure S1. ROC curves for 28- and 90-day mortality in septic shock based on the Sepsis-3 criteria.

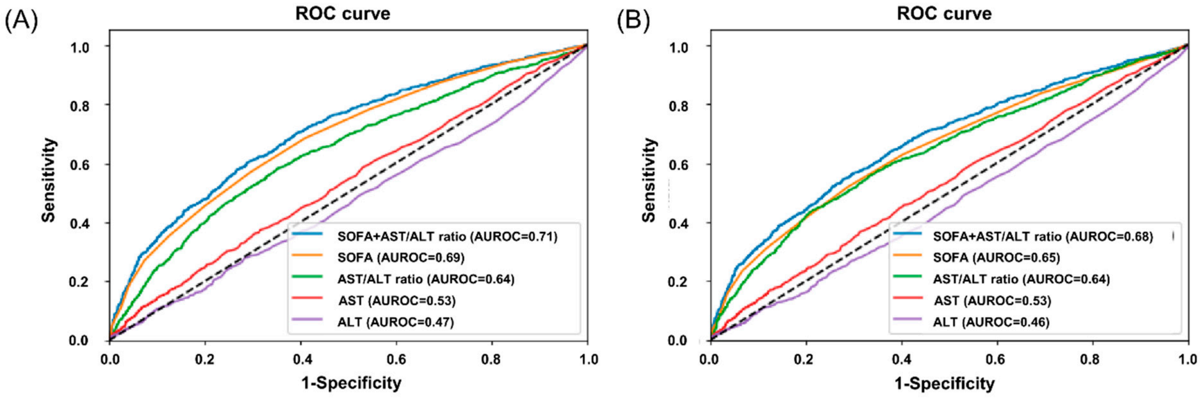

(A) ROC curves for 28-day mortality. The AUROC for SOFA + AST/ALT ratio is 0.71 (95% CI, 0.69–0.73, $p < 0.01$), SOFA is 0.69 (95% CI, 0.67–0.71, $p < 0.01$), AST/ALT ratio is 0.64 (95% CI, 0.62–0.66, $p < 0.01$), AST is 0.53 (95% CI, 0.52–0.56, $p < 0.01$), and ALT is 0.47 (95% CI, 0.44–0.51, $p = 0.78$).

(B) ROC curves for 90-day mortality. The AUROC for SOFA + AST/ALT ratio is 0.68 (95% CI, 0.66–0.70, $p < 0.01$), SOFA is 0.65 (95% CI, 0.63–0.67, $p < 0.01$), AST/ALT ratio is 0.64 (95% CI, 0.62–0.66, $p < 0.01$), AST is 0.53 (95% CI, 0.51–0.55, $p < 0.01$), and ALT is 0.46 (95% CI, 0.42–0.50, $p = 0.73$).

Figure S2. Kaplan-Meier curves for 28-day and 90-day mortality in septic shock based on the Sepsis-3 criteria.

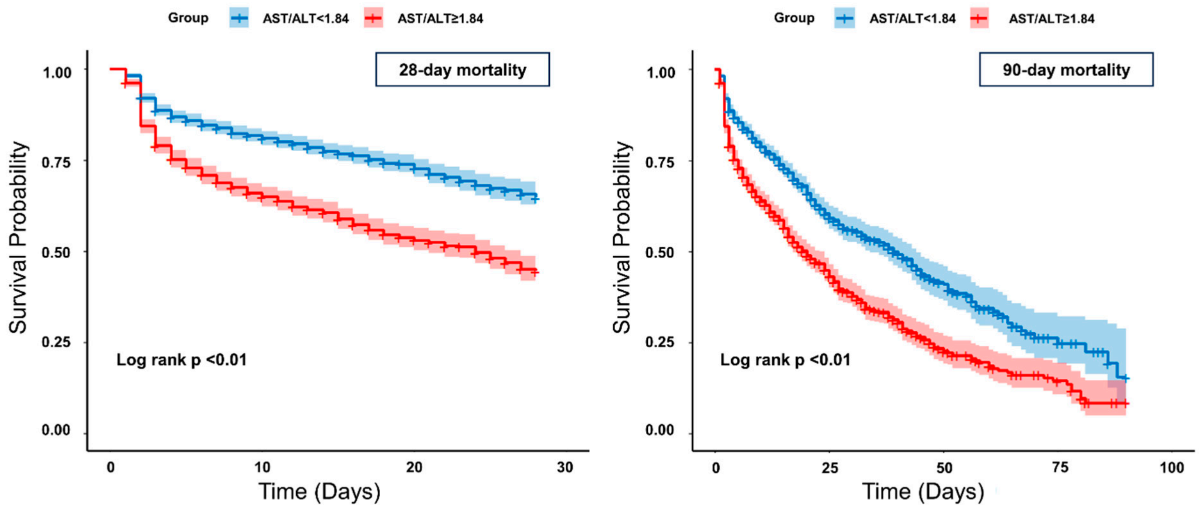

Supplement: Supplementary file 1 [file diagnostics-15-01773-s001.zip › diagnostics-3733719-supplementary.pdf]
